# Effect of a Four-Week Thrower’s Ten Exercise Program on Upper Extremity Dynamic Balance and Strength in Racket Sports Players

**DOI:** 10.7759/cureus.116390

**Published:** 2026-09-16

**Authors:** Purvi Patel, Jayesh Vaishnav

**Affiliations:** 1 Sports Physiotherapy, Sumandeep Vidyapeeth Deemed to be University, Vadodara, IND

**Keywords:** medicine ball throw test, racket sports, thrower's ten exercise, upper extremity performance, upper quarter y balance test

## Abstract

Purpose

This study aimed to evaluate the effect of a four-week Thrower’s Ten exercise program on upper extremity dynamic balance and strength in racket sports players.

Methods

A prospective single-group pre-post interventional study was conducted on 32 racket sports players (badminton and lawn tennis) aged 18-40 years, recruited through purposive sampling from sports complexes in Vadodara, India. Upper extremity dynamic balance and strength were assessed using the Upper Quarter Y Balance Test (UQYBT) and the Medicine Ball Throw Test (MBTT), respectively, before and after a four-week, three-sessions-per-week Thrower’s Ten exercise program delivered under physiotherapist supervision using progressive Theraband resistance (The Hygenic Corporation, Akron, OH, USA).

Results

UQYBT composite reach (normalized to limb length) improved significantly for the right (106.4 ± 3.8% to 114.1 ± 5.1%; mean difference, 7.69%; 95% CI, 6.83-8.55; p < 0.001) and left upper extremity (105.8 ± 3.2% to 114.8 ± 5.6%; mean difference, 8.98%; 95% CI, 7.42-10.54; p < 0.001; n = 31 owing to one missing baseline value). MBTT distance improved significantly from 23.1 ± 3.1 ft to 24.8 ± 3.4 ft (mean difference, 1.70 ft; 95% CI, 1.40-2.00; p < 0.001). Male participants demonstrated significantly greater post-intervention MBTT scores than female participants (25.73 ± 2.19 ft vs. 19.54 ± 4.05 ft; independent-samples t-test, p < 0.001), while no significant differences were observed by limb dominance, sport type, or training frequency.

Conclusions

Participants showed significant improvements in upper extremity dynamic balance and strength following the four-week Thrower’s Ten exercise program. These findings support the use of structured upper extremity strengthening programs in training routines for racket sport athletes; however, controlled trials are needed to determine whether the observed improvements are attributable to the intervention beyond natural variation or practice effects.

## Introduction

Racket sports, including badminton, lawn tennis, squash, and racquetball, are widely practiced at both recreational and competitive levels and require technical proficiency, physical fitness, and well-coordinated neuromuscular control to execute repeated overhead and arm strokes with speed and accuracy [1,2]. The serve, smash, overhead clear, and groundstroke all depend on rapid, repetitive overhead arm motion that generates high angular velocity at the shoulder and elbow while preserving racket control, and these high-speed actions transmit considerable mechanical load through the muscles, tendons, and joint structures of the upper limb [1-3]. Efficient stroke production relies on coordinated transfer of force through the kinetic chain, from the lower limbs and trunk to the upper extremity and finally to the racket; disruption at any point in this chain can increase compensatory loading on the shoulder and elbow and reduce the efficiency of force production [2,4].

Because the glenohumeral joint favors mobility over bony stability, it depends heavily on dynamic muscular support, particularly from the rotator cuff and scapular stabilizers, to maintain joint alignment during high-velocity activity [3,5]. Muscular weakness, imbalance, or fatigue can alter movement mechanics and increase stress on passive stabilizing structures such as the capsule, labrum, and ligaments, contributing to conditions including subacromial impingement, rotator cuff tendinopathy, and functional shoulder instability [3,6]. The elbow is similarly exposed to repetitive valgus stress during the acceleration and deceleration phases of overhead strokes, and adequate strength and endurance around the elbow are required to maintain joint stability under repeated loading [4,7].

Structured strengthening programs have therefore been developed to address the specific demands of overhead sport, with the primary aims of improving rotator cuff and scapular stabilizer strength, neuromuscular control, and dynamic joint stability [4,8]. Among these, the Thrower’s Ten exercise program is a widely used progressive strengthening protocol targeting the rotator cuff, deltoid, scapular stabilizers, and elbow musculature and has been extensively studied and applied in throwing athletes such as baseball players for injury prevention, rehabilitation, and performance enhancement [4,7,8].

Despite this evidence base in throwing athletes, comparatively little research has examined the effect of the Thrower’s Ten program in racket sport populations. Racket sports differ from baseball-type throwing in stroke mechanics, equipment characteristics, and movement demands; the racket itself alters the length and inertial properties of the limb, the dominant strokes (overhead clears, smashes, and serves) follow different kinematic patterns than a single overarm throw, and movement is typically repeated at much higher frequency within a rally or match than in a single throwing action [1,2,4]. Whether the functional gains demonstrated by the Thrower’s Ten program in throwing athletes generalize to racket sport players, who place different demands on the shoulder and elbow, has not been established, and no Indian data on this question currently exist. Functional outcome measures that capture both dynamic balance and explosive strength of the upper extremity, such as the Upper Quarter Y Balance Test (UQYBT) and the Medicine Ball Throw Test (MBTT), are well suited to evaluating this gap because they reflect the proprioceptive, neuromuscular, and force-generating demands placed on the shoulder during racket sport activity [9,10].

Therefore, the present study aimed to investigate the effect of a four-week Thrower’s Ten exercise program on upper extremity dynamic balance, assessed using the UQYBT, and upper extremity strength, assessed using the MBTT, in players involved in racket sports.

## Materials and methods

Study design and setting

This study was designed as a prospective, single-group, pre-post interventional study. No control or comparison group was included; all enrolled participants received the Thrower’s Ten exercise program, with outcomes compared within the same group before and after the intervention. The study was conducted in accordance with the principles of the Declaration of Helsinki. Ethical approval was obtained from the Sumandeep Vidyapeeth Institutional Ethics Committee (SVIEC/ON/Phys/BN MPT24/March/25/20), and the trial was prospectively registered with the Clinical Trials Registry - India (CTRI) (CTRI/2025/04/084562). Participant recruitment and data collection were conducted from May 2025 to March 2026. Written informed consent was obtained from all participants prior to enrollment.

Participants and sampling

Racket sports players were recruited from local sports complexes in Vadodara using purposive sampling.

Eligibility criteria

Participants were eligible if they were 18-40 years of age, male or female, had played a racket sport for at least three years for a minimum of three days per week, and were willing to participate. Participants were excluded if they had current musculoskeletal pain or injury affecting the shoulder, arm, or upper back; a neurological condition impairing motor function or coordination; a cardiovascular condition limiting participation in physical activity; sedentary status, defined as no regular physical activity, sport, or exercise in the preceding six months; any upper extremity injury within the previous six months; or any prior history of upper extremity surgery.

Participant screening and enrollment

Forty-four racket sports players were screened for eligibility. Twelve were excluded (three for recurrent shoulder dislocation, two for prior fracture of the humerus or radius/ulna, five for recent rotator cuff strain, and two for current shoulder pain), leaving 32 participants who met the inclusion criteria and were enrolled. All 32 participants completed both pre-intervention and post-intervention assessments, with no dropouts.

Sample size

The sample size was estimated a priori using the following formula:

$n = \left[\frac{Z_{\alpha/2} + Z_{\beta}}{d}\right]^2,$

where Zα/2 = 1.96 corresponds to a two-tailed significance level of 0.05, Zβ = 0.84 corresponds to 80% statistical power, and d = 0.5 represents an anticipated medium standardized effect size, calculated by hand following the conventional formula for comparing a mean difference against a fixed effect-size benchmark (Cohen’s medium effect size convention) rather than software-based estimation. This calculation yielded a minimum required sample size of 32 participants, which was the sample enrolled in this study. A medium effect size was assumed in the absence of directly comparable prior data on the Thrower’s Ten program in racket sport populations; this assumption, and the resulting sample size, should be interpreted as a pragmatic a priori estimate rather than one derived from a population-specific pilot effect size.

Outcome measures

UQYBT

The UQYBT is a dynamic, closed-chain assessment of upper extremity reach distance, stability, and neuromuscular control performed from a modified push-up position, with participants reaching the test hand sequentially in the medial, inferolateral, and superolateral directions while maintaining stance-arm and trunk stability [9]. Before formal testing, participants completed two practice trials in each reach direction for each limb. During formal testing, three trials were performed in each direction, and the maximum reach distance was recorded to the nearest 0.5 cm. A composite reach score was calculated for each limb by summing the three directional reach distances and normalizing to limb length, expressed as a percentage [9,11]. One baseline left-side UQYBT measurement was missing. This participant was excluded from the left-side pre-post analysis, and the analysis was performed using the 31 participants with complete paired measurements. The UQYBT has demonstrated good to excellent inter-rater and intra-rater reliability (intraclass correlation coefficient = 0.83-0.99) in healthy and athletic populations in previously published validation studies [9,11].

MBTT

The MBTT was used to assess upper extremity strength in a functional throwing task, with performance also influenced by rapid force generation and neuromuscular coordination. Participants stood with their feet shoulder-width apart and held a 2-kg medicine ball with both hands slightly behind the chest. They then threw the ball forward as far as possible using a movement similar to a soccer sideline throw-in. Forward stepping was permitted after release. Three trials were performed, with the best throw used for analysis. Throw distance was measured from the starting position to the point where the ball landed, to the nearest 0.5 ft. The same testing procedure was used for pre-intervention and post-intervention assessment. The MBTT has demonstrated good test-retest reliability in athletic populations [10,12].

Intervention

Following baseline assessment, participants underwent the complete Thrower’s Ten exercise program for four weeks, three sessions per week, under physiotherapist supervision. During the first week, all exercises were performed using a short lever arm and low-resistance green Theraband (The Hygenic Corporation, Akron, OH, USA), with three sets of 8-10 repetitions and a 30-second rest interval between sets. In the second week, the exercise intensity was progressed by increasing the lever arm while maintaining the same sets, repetitions, and rest interval. During the third and fourth weeks, resistance was progressed by changing from the green to a higher-resistance blue Theraband, and the press-up exercise was added to the protocol. The complete Thrower’s Ten exercise protocol was administered bilaterally. Following completion of the four-week intervention, all outcome measures were reassessed using the same procedures as at baseline [13].

Statistical analysis

Data were analyzed using SPSS Statistics version 16 (released 2007; SPSS Inc., Chicago, IL, USA) and Microsoft Excel 2007 (Microsoft Corporation, Redmond, WA, USA). Normality of the continuous outcome variables (UQYBT and MBTT, pre-intervention and post-intervention) was assessed using the Shapiro-Wilk test; all variables were normally distributed (p > 0.05), and parametric statistical tests were therefore used throughout. Descriptive statistics are reported as mean ± SD for continuous variables and as frequency (percentage) for categorical variables. Within-group pre-post differences were analyzed using the paired-samples t-test. Between-group comparisons (by gender, limb dominance, and sport type) were analyzed using the independent-samples t-test, and comparisons across training frequency subgroups were analyzed using one-way ANOVA with Tukey’s honestly significant difference (HSD) and Bonferroni post hoc tests for pairwise comparison. Effect sizes for paired comparisons are reported as Cohen’s d, calculated as the mean difference divided by the SD of the within-subject differences, together with 95% confidence intervals for the mean difference. Statistical significance was set at p < 0.05.

## Results

Forty-four racket sports players were screened, of whom 12 were excluded, and 32 met the eligibility criteria and were enrolled (Figure 1). All 32 participants completed the four-week intervention and both assessment time points, with no dropouts. Participant demographic and sport characteristics are presented in Table 1. The sample had a mean age of 25.9 ± 4.0 years (range, 19-39), was predominantly male (84%) and right-hand dominant (84%), and was composed primarily of badminton players (69%), with the remainder playing lawn tennis (31%).

**Figure 1:**
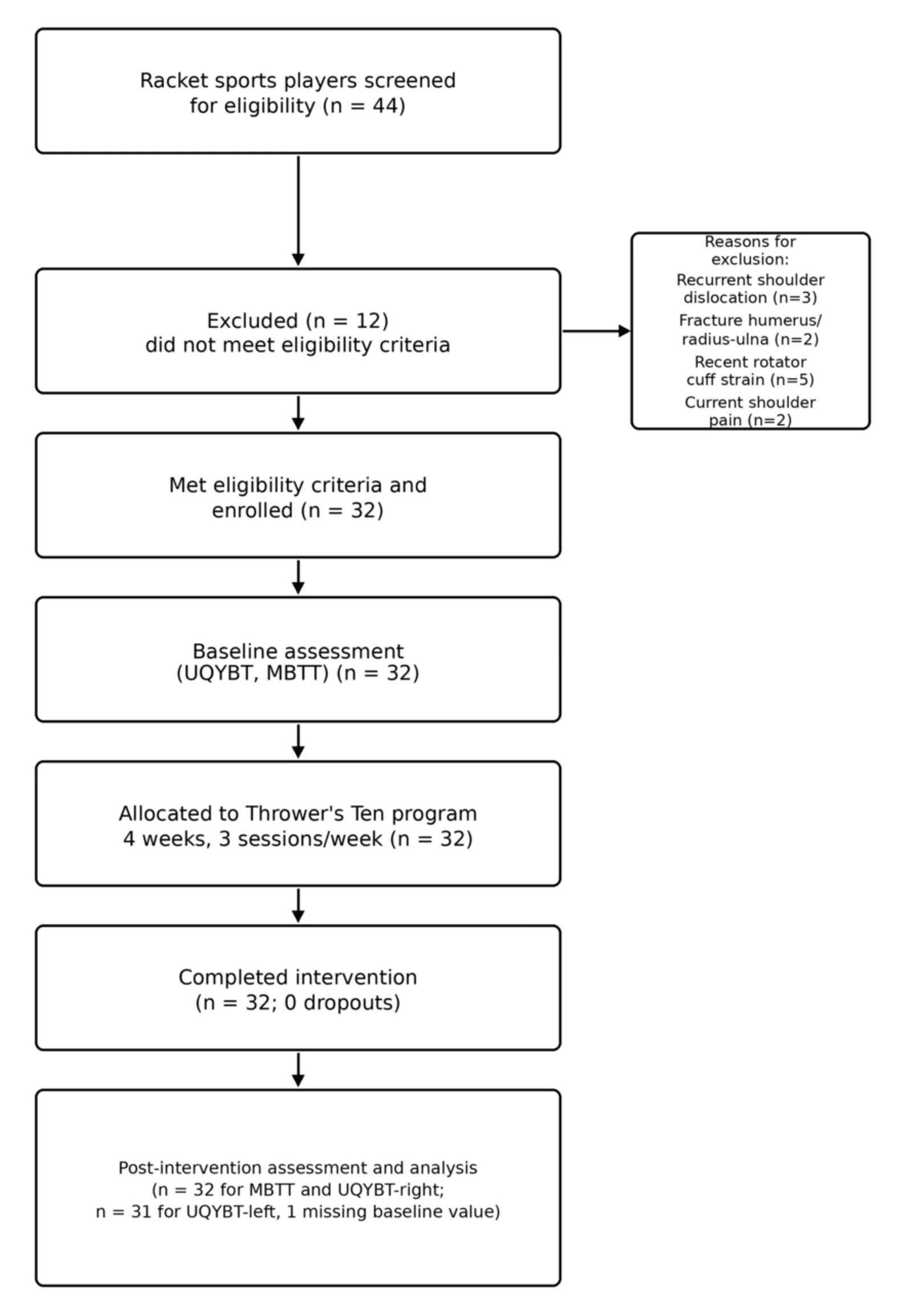
Flowchart of participant screening, enrollment, intervention, and analysis. MBTT, Medicine Ball Throw Test; UQYBT, Upper Quarter Y Balance Test

**Table 1:** Demographic and sport characteristics of participants (n = 32).

Variable	Category	n (%)
Age (years), mean ± SD	25.9 ± 4.0 (range 19-39)	-
Height (cm), mean ± SD	175.2 ± 5.2	-
Weight (kg), mean ± SD	73.1 ± 7.3	-
Gender	Male	27 (84%)
	Female	5 (16%)
Hand dominance	Right	27 (84%)
	Left	5 (16%)
Occupation	Student	22 (69%)
	Service	9 (28%)
	Coach	1 (3%)
Sport	Badminton	22 (69%)
	Lawn tennis	10 (31%)
Racket-sport training frequency (days/week)	Four days/week	5 (16%)
	Five days/week	16 (50%)
	Six days/week	11 (34%)

Within-group analysis showed statistically significant improvements in both upper extremity dynamic balance and strength following the four-week intervention (Table 2). The composite UQYBT reach score (normalized to limb length) improved significantly for the right upper extremity, from 106.4 ± 3.8% to 114.1 ± 5.1% (t(31) = 18.32, p < 0.001, mean difference, 7.69%; 95% CI, 6.83-8.55; Cohen’s d = 3.24). One participant lacked a baseline left-side UQYBT measurement; the left upper extremity analysis was therefore conducted on the remaining 31 participants and also showed a significant improvement, from 105.8 ± 3.2% to 114.8 ± 5.6% (t(30) = 11.77, p < 0.001, mean difference, 8.98%; 95% CI, 7.42-10.54; Cohen’s d = 2.11). MBTT throw distance improved significantly from 23.1 ± 3.1 ft to 24.8 ± 3.4 ft (t(31) = 11.68, p < 0.001, mean difference, 1.70 ft; 95% CI, 1.40-2.00; Cohen’s d = 2.06).

**Table 2:** Pre- and post-intervention comparison of UQYBT and MBTT scores. MBTT, Medicine Ball Throw Test; UQYBT, Upper Quarter Y Balance Test

Outcome	n	Timepoint	Mean ± SD	Mean difference (95% CI)	t (df)	p-value	Cohen’s d
UQYBT right (% limb length)	32	Pre	106.37 ± 3.82	7.69 (6.83-8.55)	18.32 (31)	<0.001	3.24
		Post	114.06 ± 5.07	-	-	-	-
UQYBT left (% limb length)	31	Pre	105.77 ± 3.19	8.98 (7.42-10.54)	11.77 (30)	<0.001	2.11
		Post	114.75 ± 5.61	-	-	-	-
MBTT (ft)	32	Pre	23.07 ± 3.09	1.70 (1.40-2.00)	11.68 (31)	<0.001	2.06
		Post	24.77 ± 3.37	-	-	-	-

Subgroup comparisons of post-intervention scores by gender, limb dominance, sport type, and training frequency are presented in Table 3. Male participants demonstrated significantly greater MBTT scores than female participants (25.73 ± 2.19 ft vs. 19.54 ± 4.05 ft; t = 5.05, p < 0.001), while no significant gender difference was observed for UQYBT scores on either side (p > 0.05). No statistically significant differences were observed by limb dominance, sport type, or training frequency for either outcome measure (all p > 0.05); pairwise post-hoc comparisons for training frequency (Tukey’s HSD and Bonferroni) likewise showed no significant differences between subgroups.

**Table 3:** Comparison of post-intervention UQYBT and MBTT scores by gender, dominance, sport type, and training frequency. MBTT, Medicine Ball Throw Test; UQYBT, Upper Quarter Y Balance Test (composite reach normalized to limb length)

Variable	Category	n	UQYBT right (%)	UQYBT left (%)	MBTT (ft)
Gender	Male	27	113.85 ± 5.30	114.54 ± 6.25	25.73 ± 2.19
	Female	5	115.16 ± 3.89	113.63 ± 3.50	19.54 ± 4.05
	p-value		0.606	0.755	<0.001
Hand dominance	Right-dominant	27	114.11 ± 5.12	114.53 ± 6.13	24.43 ± 3.40
	Left-dominant	5	113.80 ± 4.71	113.70 ± 4.71	26.58 ± 2.85
	p-value		0.902	0.777	0.195
Sport type	Badminton	22	114.13 ± 5.46	113.84 ± 5.40	24.95 ± 3.27
	Lawn tennis	10	113.90 ± 4.37	115.63 ± 6.94	24.36 ± 3.73
	p-value		0.906	0.432	0.654
Training frequency	Four days/week	5	112.87 ± 3.61	112.94 ± 3.93	24.18 ± 3.32
	Five days/week	16	114.49 ± 5.47	115.07 ± 6.64	24.66 ± 3.70
	Six days/week	11	115.30 ± 6.94	115.48 ± 7.33	26.38 ± 2.22
	p-value		0.615	0.603	0.489

## Discussion

This study investigated the effect of a four-week Thrower’s Ten exercise program on upper extremity dynamic balance and strength in racket sports players, using the UQYBT and the MBTT. Both outcome measures showed statistically significant and, by conventional benchmarks, large within-group improvements following the intervention, consistent with the hypothesis that a structured rotator cuff and scapular stabilizer strengthening program can enhance upper extremity functional performance in this population.

Repetitive overhead strokes in badminton and lawn tennis place substantial demand on the rotator cuff, scapular stabilizers, and surrounding soft tissue, and dynamic shoulder stability depends on the integration of coordinated muscle activation with proprioceptive feedback [3,5]. The improvement observed in UQYBT performance in the present study, which assesses dynamic balance, proprioception, and neuromuscular control of the upper extremity under closed-chain loading, is consistent with the Thrower’s Ten program’s emphasis on strengthening the rotator cuff and scapular stabilizers to support glenohumeral stability during functional tasks [4,8]. These findings are in line with previous reports that the Thrower’s Ten program improves upper extremity functional performance and neuromuscular control in overhead athletes [14] and that comparable structured strengthening programs improve functional stability and biomechanical performance during overhead movement in other overhead-sport populations [15,16].

The improvement observed in MBTT performance reflects enhanced upper extremity strength. The Thrower’s Ten program specifically targets the rotator cuff, deltoid, scapular stabilizers, and forearm musculature that contribute to force generation and kinetic chain coordination during ballistic upper extremity movement [4,7,8], and resistance-based upper extremity training of this kind has previously been shown to improve shoulder strength and throwing-related performance in other athletic populations [17,18].

Subgroup analyses showed that male participants achieved significantly higher post-intervention MBTT scores than female participants, consistent with well-documented sex differences in upper body muscle mass and force production capacity [19]; by contrast, no significant gender difference was observed for UQYBT performance, suggesting that the balance and neuromuscular control demands assessed by this test may be less dependent on absolute strength than the ballistic throwing task captured by the MBTT. No significant differences were observed by limb dominance for either outcome measure. However, this finding should not be interpreted as evidence of equivalence between dominant and non-dominant limbs, particularly given the relatively small subgroup sizes [20]. Similarly, no significant differences were observed between badminton and lawn tennis players. However, this finding should be interpreted cautiously given the relatively small subgroup sizes and may reflect limited statistical power to detect true between-sport differences [1,2]. No significant differences in outcomes were observed across participants according to their usual racket-sport training frequency of four to six days per week. This frequency reflected their background racket-sport participation, whereas the Thrower’s Ten intervention was provided at a fixed frequency of three sessions per week for four weeks. Therefore, the four to six days/week variable should not be interpreted as a dose-response measure of the Thrower’s Ten intervention.

These findings should be interpreted in light of the absence of a control group. Because all participants received the intervention without a comparison group performing no intervention or an alternative intervention, the improvements observed cannot be fully attributed to the Thrower’s Ten program itself; some portion of the change may reflect natural variation, a learning or practice effect on the outcome measures themselves (particularly plausible for the UQYBT, which is a motor-skill-dependent test), or regression to the mean. The findings are best interpreted as evidence that meaningful improvement occurred over the study period in this single-group design, rather than as direct evidence of the Thrower’s Ten program’s effect relative to no intervention or to an alternative training program.

Limitations

The relatively small sample size and purposive sampling from local racket-sport settings may limit the generalizability of the findings. Additionally, the study was conducted in badminton and lawn tennis players, which may limit the applicability of the findings to athletes participating in other racket sports.

## Conclusions

A four-week Thrower’s Ten exercise program was associated with significant improvements in upper extremity dynamic balance and strength in racket sports players. These findings support the use of structured upper extremity strengthening programs in training routines for racket sport athletes. However, controlled trials are needed to determine whether the observed improvements are attributable to the intervention beyond natural variation or practice effects.
